# Barriers and facilitators related to the uptake of four strategies to prevent neonatal early-onset group B haemolytic streptococcus disease: a qualitative study

**DOI:** 10.1186/s12884-017-1314-8

**Published:** 2017-05-09

**Authors:** Diny G. E. Kolkman, Margot A. H. Fleuren, Maurice G. A. J. Wouters, Christianne J. M. de Groot, Marlies E. B. Rijnders

**Affiliations:** 1 Department of Child Health, TNO, PO Box 3005, 2301 DA Leiden, The Netherlands; 2 Department of Obstetrics and Gynaecology, VUmc, VU University Medical Centre, PO Box 7057, 1007 MB Amsterdam, The Netherlands

**Keywords:** Determinant analysis, Early-onset group B streptococcus, Guidelines, Implementation, Prevention

## Abstract

**Background:**

Actions to prevent early onset disease in neonates are based on different strategies including administering antibiotic prophylaxis during labour in case of 1) maternal GBS colonisation (screening strategy), 2) identified risk factors (risk-based strategy) or 3) a combination of these two conditions (maternal GBS colonisation and identified risk factors: combination strategy and the Dutch guideline). Low adherence to guidelines preventing EOGBS has been reported. Each strategy has drawbacks and clinical outcomes are affected by care providers’ and women’s adherence. The actual impact of any preventive strategy is the product of efficacy of the strategy and the level of implementation. In order to reduce neonatal death due to EOGBS by developing the optimal guideline, we analysed barriers and facilitators of current used strategies.

**Methods:**

Focus group and personal interviews with care providers and women were performed. Impeding and enhancing factors in adherence to the preventive strategies were discussed and scored using the Measurement Instrument for Determinants of Innovations (MIDI) and analysed by two independent researchers.

**Results:**

Overall, care providers identified 3.6 times more factors that would impede (*n* = 116) rather than facilitate (*n* = 32) adherence to the preventive strategies. 28% facilitative factors were reported in relation to the combination strategy and 86% impeding factors in relation to the Dutch guideline. The most preferred strategy was the combination strategy by 74% of the care providers and by 86% of the women.

**Discussion:**

We obtained a detailed understanding of factors that influence adherence to preventive strategies.

This insight can be used to develop implementation activities to improve the uptake of new strategies.

**Trial registration:**

The trial is registered in the Dutch Trial Register NTR3965.

**Electronic supplementary material:**

The online version of this article (doi:10.1186/s12884-017-1314-8) contains supplementary material, which is available to authorized users.

## Background

Although early-onset group B haemolytic streptococcus disease (EOGBS) is rare (incidence of 0.019 [1]−0.0.043 [2]%), it remains an important cause of perinatal mortality and long-term morbidity for the neonate and child [3, 4]. The main cause of neonatal infection in the first week of life is by vertical transmission of the group B haemolytic streptococcus (GBS) during delivery. Primary prevention of EOGBS is achievable with intra-partum antibiotic prophylaxis (IAP) for women who are GBS carriers [5].

Different preventive strategies are currently used based on identifying women at risk, either by screening for GBS colonisation and/or by identifying risk factors for EOGBS. Risk factors include a previous child with EOGBS and risk factors in the current pregnancy including group B streptococcal (GBS) bacteriuria, intra-partum fever (temperature of or over 38.0 °C), preterm birth (defined as before 37 weeks) and/or pre-labour rupture of membranes (more than 18 hours).

The four different preventive EOGBS strategies are (Table 1):

1. The screening strategy, in which a vaginal rectal swab to detect GBS is taken at 35–37 weeks of gestation. All GBS-colonised women or women with unknown GBS status receive IAP. This strategy is not used in the Netherlands.
2. The risk-based strategy. All women with one or more of the risk factors mentioned above receive IAP.
3. The combination strategy, in which a vaginal rectal swab to detect GBS is taken at 35–37 weeks of gestation. Women with both conditions: GBS colonisation and one or more of the above mentioned risk factors are treated with IAP, women with GBS colonisation without a risk factor are not treated with IAP.
4. The Dutch guideline, based on the risk-based strategy prescribes IAP for women with a previous child with EOGBS, group B streptococcal bacteriuria during current pregnancy (only determined in symptomatic women) or intra-partum fever [6]. Vaginal rectal swabs to detect GBS (by culture) are taken in case of preterm birth or pre-labour membrane rupture and IAP is given if GBS colonisation is established. If no test result is available during labour application of IAP is predominantly based on clinical observation including fever.

**Table 1:** Key activities or recommendation in the four preventive EOGBS strategies

Key activities or recommendation	Screening strategy	Risk-based strategy	Combination strategy	Dutch guideline
Identify risk factors^a^	No	Yes	Yes	Yes
Take swab at 35–37 weeks of gestation	Yes	No	Yes	No
Take swab at onset or during labour	No	No	No	All women with risk factors 4 or 5^a^
Treatment of the woman with IAP	All women who are colonised	All women with ≥ one risk factor	All women with ≥ one risk factor AND who are colonised	All women with risk factors 1, 2, or 3 All women with risk factors 4 or 5^a^ AND who are colonised
Antibiotic treatment of the child	All baby’s with signs of neonatal infection	All baby’s with signs of neonatal infection	All baby’s with signs of neonatal infection	All baby’s with signs of neonatal infection

^a1^Previous child with EOGBS, ^2^GBS bacteriuria in current pregnancy, ^3^Intra partum fever (≥38 °C), ^4^Preterm birth (<37 weeks), ^5^Pre-labour rupture of membranes (>18 hours)

However, none of the preventive strategies result in the complete prevention of EOGBS [7]. Strategies are not fully effective by themselves and have drawbacks which may result in non-adherence by both care providers and women. For example: over 40% of neonates who develop EOGBS are born to mothers without a risk factor [8, 9] (risk-based strategy, combination strategy and Dutch guideline), the sensitivity of the methods to detect GBS in pregnant women is low and accounts for a consistent proportion of EOGBS cases [9–12] (risk-based strategy, screening strategy and the Dutch guideline), large numbers of women receiving antibiotics (screening strategy) with possible negative side-effects such as antibiotic resistance [9] (based on the difference between application of antibiotics to all GBS colonized women or to a selection of GBS colonized women with a risk factor) and premature cases are missed, because the current GBS screening starts from 35 weeks of gestation onward (screening strategy and combination strategy). Another problem with low adherence to the preventive strategies is that a substantial proportion of women will not receive the intended care or even receive unnecessary care and this affects outcomes in women and their children [13].

From a socio-economic perspective the cost-effectiveness of the four preventive strategies was compared in a theoretical model in 2005 [14] assuming 100% adherence by care providers and women. The risk-based strategy and the combination strategy had the most favourable cost-effectiveness ratios; the screening strategy was the most clinically effective, but the least cost-effective. However, low adherence to guidelines preventing EOGBS is reported elsewhere and there seems to be a need for strategies to improve adoption of EOGBS guidelines [7, 15, 16]. The actual impact of any preventive strategy is the product of efficacy of the strategy and the level of implementation.

The actual implementation of innovations, (in this study the different preventive strategies perceived as new by the care providers and women) is maximised if they are introduced systematically [17–20]. Determinants affecting the implementation of innovations can be broken down depending on their association with a. preventive strategy (e.g. procedural clarity), b. the potential user of the preventive strategy (e.g. knowledge), c. the organisational context of the user (e.g. financial resources, staff turnover) and d. the socio-political context (e.g. legislation) [18, 21]. A detailed understanding of these determinants can guide the process of designing implementation strategies that will have the potential to produce actual change [18–20].

The aim of this study (part of a larger study that examines the most cost-effective prevention strategy on the basis of efficacy and feasibility in daily practice) is to assess the determinants that will influence the implementation of four preventive strategies for EOGBS among care providers and women [22].

## Methods

### Setting

The Dutch maternity care system uses a stratified care model with different professional care providers at different risk levels. Midwives working in primary care are the main group of caregivers in low-risk pregnancies. Obstetricians and hospital based midwives take care of medium- or high risk pregnancies. The agreements for collaboration between the professional groups have been specified in the Obstetric Manual [23]. This document includes a list of obstetric indications for referral from primary to secondary care setting. Care path ways are organized in Obstetric Collaboration Groups (OCGs). An OCG is organized around a hospital and consists of midwives, obstetricians and pediatricians. The OCGs make agreements about the regional organization of maternity care and interdisciplinary collaboration.

### Participants and design

Determinants of the uptake of the strategies were studied in both care providers and women (pregnant and postpartum). In case of the Dutch guideline these were perceived determinants, compared to anticipated determinants in the other strategies. OCGs in the Netherlands were asked to participate in a focus group interview. Microbiologists were interviewed in person on specific topics considering the logistics of culture sampling. Women were recruited in midwifery practices throughout the Netherlands by their midwives and the Dutch foundation parents of group B streptococcus patients (OGBS) was approached by one of the researchers.

Semi-structured interviews were chosen to let the participants talk frankly. The main topics for the care provider interviews were: a. screening for GBS colonization or identifying risk factors, b. treatment of the woman, c. treatment of the child and d. the most preferred strategy (Additional file 1). The key activities in all preventive strategies (Table 1) guided the content of the interviews, supported by a list of potentially relevant determinants (associated with the preventive strategy, the care provider or woman, the organizational context and the socio-political context) [18, 21]. The main topic for the interviews with women were: a. the most preferred strategy, b. preferences in taking a swab, c. information to be provided about GBS, d. impact of test results on anxiety, preferred place of birth and observation of the child (Additional file 2).

Prior to the interviews care providers and women received information on the different preventive strategies and the interview questions by letter or e-mail.

Two moderators guided the group interviews. The individual interviews were conducted by one moderator. The interviews continued until no additional information was gathered during subsequent interviews. All interviews were audio taped and summarized in a report which was depersonalized. The report was sent to the participants to verify and add information.

### Analysis

The transcribed interviews of the care providers and women were subjected to deductive analysis. The first focus group interview with care providers generated a list of quotations of impeding and enhancing determinants. This list was extended with new mentioned determinants in subsequent interviews. All identified impeding or enhancing factors were scored according to 29 determinants described in the Measurement Instrument for Determinants of Innovations [21] and categorised in the four domains: the guideline, the intermediary user (the care provider), the organisational context and socio-political context.

All interviews were independently analysed by two researchers. Inconsistencies were resolved by consensus and (if necessary) by consulting a third person. The interviews with women were analysed alike and identified impeding and enhancing factors which could not be categorised in the four domains mentioned above. A fifth domain was added: determinants related to the end-user (the woman).

## Results

### Participants

Five focus group interviews (four with care providers and one with women) and 12 personal interviews (two with care providers and 10 with women) were held in the period June until October 2011.

Twenty-seven care providers from different settings were interviewed in the focus group meetings which were held in four regions in the Netherlands, the participating care providers and their settings can be seen in Table 2. Two microbiologists were interviewed in person.

**Table 2:** Overview of attending care providers in focus group interviews per region

	Region 1	Region 2	Region 3	Region 4	Total
Primary care midwife	1	4	1	1	7
Hospital based midwife	2	1	1	2	6
Obstetrician in training (resident)	2	0	2	1	5
Obstetrician	1	1	1	1	4
Pediatrician	1	1	0	1	3
Obstetric nurse	0	1	0	1	2
Total	7	8	5	7	27

Fourteen women from different parts of the Netherlands were interviewed; ten were interviewed in person and four attended a focus group meeting. Seven women were of Dutch origin. The other countries of origin were Hungary, Bosnia, Morocco, Suriname and Turkey. The educational level of seven women was high, of five women medium/low and of two women unknown. One woman was not pregnant, but mother of a child with long term EOGBS complications. The median week of gestation of the pregnant women was 34 (range: 26–38); eight women were nulliparous and six multiparous of which one had an experience with GBS in a previous pregnancy. Seven women preferred a home birth, five preferred a hospital birth and of two this was unknown.

### Determinants mentioned by the care providers

The determinants associated with screening for GBS colonization or identifying risk factors (Table 3), treatment of the woman (Table 4) and treatment of the child (Table 5) show a wide variety between the four strategies. Looking at all three tables together, most determinants were reported for the combination strategy (*n* = 53) and the screening strategy (*n* = 49), followed by the risk-based strategy (*n* = 25) and the Dutch guideline (*n* = 21). The determinants were 3.6 times more often (116 times) reported as impeding than they were reported as facilitating (32 times). Most determinants were associated with the user (*n* = 71), followed by the guideline itself (*n* = 39), the organisational context (*n* = 34) and the socio-political context (*n* = 4).

**Table 3:** Determinants related to identifying risk factors or screening for GBS colonization, mentioned by care providers (*n* = 25)

Key activities	Determinants	Screening strategy ^1.2^	Risk-based strategy^1,2^	Combination strategy^1,2^	Dutch guideline ^1,2^
**Identifying risk factors**	Procedural clarity (guideline)	3	3	3	3
	Unclear definition previous child with EOGBS	*N*	*N*	*N*	*N*
	No standard cut-off point urinary tract infection despite guideline	*N*	*N*	*N*	*N*
	No standard procedure PROM (referral after 18, 24, > 24 hours)	*N*	*N*	*N*	*N*
	Correctness (guideline)	1	1	1	1
	Symptoms of urinary tract infection are often missed	*N*	*N*	*N*	*N*
	Social support by other care providers (user)	2	2	2	2
	No adequate history taking of previous child with EOGBS	*N*	*N*	*N*	*N*
	Not standard GBS detection in urine culture by general practitioner, therefore AB treatment not directed at GBS because status unknown	*N*	*N*	*N*	*N*
	Legislation and regulations (socio-political context)	1	1	1	1
	Data exchange between care providers of previous pregnancy	*N*	*N*	*N*	*N*
**Screening for GBS colonization (swab taking)**	Procedural clarity (guideline)	1	0	1	1
	Local differences in swab taking (vaginal /vs vaginal rectal /vs urine)	*N*		*N*	*N*
	Correctness (guideline)	0	0	0	1
	Test result swab taken during birth not available in time				*N*
	Compatibility with current guideline (guideline)	1	0	1	0
	Collaboration with laboratory already in place	*P*		*P*	
	Personal benefits / drawbacks (user)	1	0	1	0
	Extra work for primary care midwives	*N*		*N*	
	Outcome expectations (user)	4	0	4	0
	Sensitivity/specificity swab not 100% because of intermittent carrier status	*N*		*N*	
	Women can adequately take swab themselves (validity culture)	*N/P*		*N/P*	
	Swab result not available for every woman at time of birth	*N*		*N*	
	Client/patient satisfaction (user)	3	0	3	0
	Women do not like swab taking	*N*		*N*	
	Women prefer swab taking for reassurance	*P*		*P*	
	Increases anxiety in women and partners	*N*		*N*	
	Social support by other care provider (user)	2	0	2	0
	Data exchange culture results	*N/P*		*N/P*	
	Self-efficacy (user)	1	0	1	0
	Confidence in discussing test results with women	*P*		*P*	
	Knowledge (user)	1	0	1	0
	Care providers know how to take a swab	*P*		*P*	
	Time available (organisational context)	1	0	1	0
	Time consuming because of providing information and swab taking	*N*		*N*	
	Staff capacity (organisational context)	1	0	1	0
	Sufficient capacity laboratory personnel	*P*		*P*	
	Material resources and facilities (organisational context)	3	0	3	0
	Easy to administer in primary care	*P*		*P*	
	Swabs not available	*N*		*N*	
	Often delay if culture taken in primary care	*N*		*N*	
	Financial resources (organisational context)	2	0	2	0
	Increased costs in primary care (swab taking)	*N*		*N*	
	Increased costs in laboratory personnel	*N*		*N*	
	Total numbers of determinants, related to	28	7	28	9
	the guideline itself	6	4	6	6
	the user (care provider)	14	2	14	2
	the organisational context	7	0	7	0
	the socio-political context	1	1	1	1

^1^ ‘*P*’ = positive determinant; ‘*N*’ = negative determinant; ‘*N/P*’ = both negative and positive determinant. ^2^ The numbers in each row reflect how many issues related to the determinant were mentioned by the care providers

**Table 4:** Determinants related to treatment of the woman, mentioned by care providers (*n* = 25)

Key activities	Determinants	Screening strategy ^1.2^	Risk-based strategy^1,2^	Combination strategy^1,2^	Dutch guideline ^1,2^
**Antibiotic prophylaxis in the woman**	Procedural clarity (guideline)	0	0	1	1
	Logistics birth at home GBS positive mother without risk factor			*N*	
	No standard treatment in case of PROM/ –preterm birth and unknown results swab				*N*
	Complexity (guideline)	0	1	0	0
	Easy to follow		*P*		
	Compatibility (guideline)	0	1	0	1
	Is already in practice		*P*		*P*
	Outcome expectations (user)	4	3	6	0
	Over treatment (IAP, hospital birth, observation baby)	*N*	*N*	*N*	
	No over treatment	*N*		*P*	
	Under treatment	*N*	*N*		
	Increase AB resistance problem			*N*	
	No increase AB resistance problem			*P*	
	More tailored care in case of GBS carrier ship and PROM	*N*		*P*	
	Increase hospital birth/decrease home birth		*N*	*N*	
	Personal benefits / drawbacks (user)	0	1	1	0
	No extra work for primary care midwives		*P*	*N*	
	Client/patient satisfaction (user)	0	0	2	0
	GBS positive without risk factor: AB prophylaxis desired by woman			*N*	
	Suits women critical of AB prophylaxis			*P*	
	Staff capacity (organisational context)	1	1	1	1
	Enough capacity in hospital	*N*	*N*	*P*	*P*
	Material resources and facilities (organisational context)	1	2	1	2
	No problem in daily practice		*P*		*P*
	Penicillin not always available in hospital because of pharmacy policy	*N*	*N*	*N*	*N*
	Financial resources (organisational context)	1	0	1	0
	No reimbursement	*N*		*N*	
	Total numbers of determinants, related to	7	9	13	5
	the guideline itself	0	2	1	2
	the user (care provider)	4	4	9	0
	the organisational context	3	3	3	3
	the socio-political context	0	0	0	0

^1^ ‘*P*’ = positive determinant; ‘*N’* = negative determinant; ‘*N/P*’ = both negative and positive determinant. ^2^ The numbers in each row reflect how many issues related to the determinant were mentioned by the care providers

**Table 5:** Determinants related to treatment of the child, mentioned by care providers (*n* = 25)

Key activities	Determinants	Screening strategy ^1.2^	Risk-based strategy^1,2^	Combination strategy^1,2^	Dutch guideline ^1,2^
**Treatment (AB) and observation of the child**	Procedural clarity (guideline)	4	2	4	2
	Logistics observation child at home GBS positive mother without risk factor	*N/P*		*N/P*	
	AB treatment differs between 3 to 5 days treatment	*N*	*N*	*N*	*N*
	AB prophylaxis preterm children not specified	*N*	*N*	*N*	*N*
	Personal benefits/drawbacks (user)	1	0	1	0
	Resistance hospital staff admission child and mother after birth	*N*		*N*	
	Outcome expectations (user)	6	4	4	2
	40% of cases are missed	*N*	*N*	*N*	*N*
	Increase in yield infection	*N*	*N*		
	AB resistance problem	*N*	*N*		
	Mother or postpartum nursing-aid can adequately observe the child at home of GBS positive mother without risk factor	*N/P*		*N/P*	
	Hospital not always safe for observation child	*N*	*N*	*N*	*N*
	Knowledge (user)	1	1	1	1
	Postpartum nursing-aid needs training because of insufficient knowledge	*N*	*N*	*N*	*N*
	Financial resources (organisational context)	1	1	1	1
	Increased costs because of culture taking in the child	*N*	*N*	*N*	*N*
	Time available (organisational context)	1	1	1	1
	Taking cultures in a child is time consuming	*N*	*N*	*N*	*N*
	Total numbers of determinants, related to	14	9	12	7
	the guideline itself	4	2	4	2
	the user (care provider)	8	5	6	3
	the organisational context	2	2	2	2
	the socio-political context	0	0	0	0

^1^ ‘*P*’ = positive determinant; ‘*N’* = negative determinant; ‘*N/P*’ = both negative and positive determinant. ^2^ The numbers in each row reflect how many issues related to the determinant were mentioned by the care providers

Because key activities were discussed in detail, it became apparent that some participants were not fully aware about the content of the Dutch guideline and many recommendations in the current guideline lacked procedural clarity. For example, the definition of a previous child with EOGBS was unclear and there was no standard procedure for pre-labour rupture of membranes (referral after 18, 24, or more than 24 hours). It also became apparent that the guideline was not fully adhered to in many cases, due to other determinants as described in the Tables 3, 4, and 5.

Looking closely at the determinants mentioned for screening or identifying risk factors (Table 3), it becomes clear that care providers acknowledged the limitations of a swab taken during delivery. They did not anticipate logistic problems taking a swab in pregnancy although they expected it to be time-consuming, specifically because of anticipated anxiety by women. Care providers expected that although women might prefer the assurance of a swab taken they would dislike it because of discomfort.

With respect to treatment of the woman, Table 4 shows that routine antibiotics in case of carrier status was expected to result in overtreatment, negative side effects such as an increase in antibiotic resistance, medicalization of birth and a decrease of the choices of women to opt for home birth.

As for the treatment of the child (Table 5) care providers differed in their opinion on the duration of and preferred place for observation of a child after a risk factor or GBS carrier status of the mother.

After having discussed the four strategies in detail, 74% (20 of 27) of the care providers felt that the combination strategy would be the most preferred strategy to be introduced. According to the care providers this strategy has the optimum in numbers needed to treat and is the most cost-effective one. The screening strategy was seen as most effective in preventing EOGBS. However, it was the least favorite because of the expected negative side effects, overtreatment and increase in antibiotic resistance (highest numbers needed to treat). Most of the interviewed care providers (89%, 24 of 27) advised negatively on the implementation of this strategy.

### Determinants mentioned by the women

Asked for the strategy the women preferred most, 12 (12 of 14, 86%) women said the combination strategy; one woman (1 of 14, 7%) preferred the Dutch guideline and one (7%) the screening strategy. Three women (3 of 14, 21%) who preferred the combination strategy considered the Dutch guideline second best. Four women (4 of 14, 29%) spontaneously mentioned not to participate in the screening strategy. The main reasons for choosing or declining a strategy were: risk of over treatment (*n* = 13 of 14, 93%), negative effects for the woman or the child (*n* = 3, 21%), ability of early treatment in case of GBS positive woman (*n* = 1, 7%) and costs (*n* = 1, 7%).

The vast majority of women (*n* = 13, 93%) had no objection at all to taking a swab and were even prepared to pay for the test themselves, if necessary. A swab taken by the professional was the primary choice of most women (*n* = 12 of 14, 86%), because of the expected better outcomes and physical difficulties if performed by themselves.

Regarding the information to be provided about GBS, most women (n = 13, 93%) wanted to receive general information about GBS in the first half of the pregnancy, preferably through a leaflet or on a website. Being personally informed was not deemed necessary. However, positive test results should be communicated in person (*n* = 14, 100%).

Five women (5 of 14, 36%) anticipated anxiety after a positive test result. Eight women (8 of 14, 57%) did not anticipate anxiety because treatment is possible and the risk of a sick child was estimated to be low. The women expected their preferred place of birth not to change because of a GBS carrier status. Five women (36%) expressed explicitly that the choice for place of birth should always be the woman’s decision. The preferred place for observing the child in case of GBS carrier status without a risk factor was in almost all cases equal to their preferred place of birth. Women expressed faith in their care providers to guide them adequately. Women who preferred their child to be observed at home also expressed that they were capable to do so and could take responsibility.

## Discussion

The present study shows that many determinants are anticipated to influence the implementation of preventive strategies for EOGBS. Overall respondents identified more factors that would impede rather than facilitate their introduction. Care providers and women did basically not differ with respect to the most or less preferred strategy, but they unanimously felt the screening strategy should not be introduced because of the expected negative side effects, i.e. overtreatment and increase in antibiotic resistance. In the Netherlands, there is a restrictive policy regarding application of antibiotics. Application of the screening strategy increases the exposure to antibiotics. However, care providers and women have a different view on some determinants. Care providers anticipated non-cooperation from women in taking a swab because women will dislike this, while nearly all women reported to have no problem at all with taking a swab. Furthermore, care providers assume the test results will increase anxiety in women. This is certainly not applicable for all the interviewed women.

Also, impeding determinants were reported in adherence to the Dutch guideline. It is noteworthy that care providers deviated from the key recommendations in many cases either because they were not familiar with them, or because they interpreted the recommendations in their own way in case of procedural unclarity. This means that the uptake of the current guideline could benefit from new implementation activities. If knowledge on the specifics of the guidelines (different strategies) is low and no uniformity in the utilization of the guidelines is achieved prevention of EOGBS will not increase.

Our study is limited in some respects. The numbers of care providers and women we interviewed are relatively small. Yet, data saturation was reached during the last interviews suggesting these numbers are sufficient. This does not rule out that the respondents may not have been representative of all care providers or women.

Our research gives insight in the determinants that affect the uptake of the preventive strategies, in 48% associated with the user (e.g. knowledge) and in 26% with the guideline itself (e.g. procedural clarity). Since the study is part of a larger study with the aim to examine the most cost-effective prevention strategy on the basis of both efficacy and feasibility in daily practice [22], the next step is to accommodate these determinants into implementation strategies. Looking at the determinants that are associated with the preventive strategies, we must first clarify some recommendations and provide answers to assumed incorrectness of other recommendations. Furthermore, we will fine-tune the patient brochures that we used in the present study for providing information about the preventive strategies.

Determinants related to the care provider will be addressed in a training that is ‘obliged’ for all care providers in the pilot regions. This training will focus on the knowledge and skills needed to be able to perform the allocated preventive strategy.

In general, determinants related to the organisational or socio-political context are more difficult to change. Looking at the determinants mentioned in the present study, some can be solved by redesigning the logistics of the care process in the hospital. Others can be solved by making clear agreements in the OCG between all care providers, such as on timely data exchange. Determinants like reimbursement or available time are difficult to address within the project, but will be part of the cost-effectiveness study.

## Conclusion

In summary, this study gives a detailed understanding of the determinants that influence adherence to the Dutch guideline and the anticipated determinants if the screening strategy, risk-based strategy or the combination strategy are introduced. As both the care providers and women advised negatively, the screening strategy is not likely to be introduced. The preventive strategies will be introduced and studied in three pilot regions; we will adapt the implementation activities for the introduction of the strategies according to the specific determinants found in the present study.

## Additional files

Additional file 1: The interview questions for the focus groups with care providers. (DOCX 14 kb)

Additional file 2: The interview questions for the focus group or individual interviews with women. (DOCX 14 kb)

## Abbreviations

AB: Antibiotics
EOGBS: Early-onset group B haemolytic streptococcus
IAP: Intra-partum antibiotic prophylaxis
GBS: Group B haemolytic streptococcus
OCG: Obstetric Collaboration Group
OGBS: Foundation parents of group B streptococcus patients
PROM: Pre-labour rupture of membranes
