# Supplementary material for: Barriers and facilitators related to the uptake of four strategies to prevent neonatal early-onset group B haemolytic streptococcus disease: a qualitative study
Source: BMC Pregnancy Childbirth. 2017 May 9;17:139. doi: 10.1186/s12884-017-1314-8 (PMC5423122; doi:10.1186/s12884-017-1314-8)
Supplement: Supplementary file 1 — The interview questions for the focus groups with care providers. (DOCX 14 kb) [file 12884_2017_1314_MOESM1_ESM.docx]

Questions for the focus group interviews with professionals

1. How do feel about the four different preventive strategies: the current method (Dutch guideline), the risk-based strategy, the screening strategy and the combination strategy? Are the strategies acceptable for you or not and why do you feel this way?
2. Which implementation problems do you foresee in daily practice? Which solutions can you think of? *Consider: maternal screening logistics, consultation general practitioner/ midwife/ gynaecologist, logistics regarding the use of chlorhexidine*

2a. Which problems relate to you as a professional? *Consider: the relevance of the preventive strategy for you (personally), knowledge and skills you need to perform the strategy (explaining the different strategies, for example).*

2b. Which problems relate to your organisation? *Consider support of colleagues, workload, available time, availability of leaflets.*

2c. Which problems relate to the broader environment*? Consider consultation with care providers in the obstetric collaboration, wishes and cooperation of parents.*

1. Do you think women will accept the policy of maternal screening and administration of antibiotics during labour as described in the different strategies? What are the reasons not to accept the policy? Which possible solutions can you think of? *Consider: choice of place of birth for women receiving care from primary-care midwives, chlorhexidine flushing, treatment with antibiotics, a risk factor in history and a negative maternal swab result (no GBS colonisation detected).*
2. Is there a strategy which you would definitely advise against in the context of the study? If so, which strategy and why?
3. What should we do or avoid in order to optimise the strategies during the study? *Consider: logistics, materials to inform/educate pregnant women, training for care providers, organisation, cooperation in the obstetric collaboration groups.*
