# Supplementary material for: Barriers and facilitators related to the uptake of four strategies to prevent neonatal early-onset group B haemolytic streptococcus disease: a qualitative study
Source: BMC Pregnancy Childbirth. 2017 May 9;17:139. doi: 10.1186/s12884-017-1314-8 (PMC5423122; doi:10.1186/s12884-017-1314-8)
Supplement: Supplementary file 2 — The interview questions for the focus group or individual interviews with women. (DOCX 14 kb) [file 12884_2017_1314_MOESM2_ESM.docx]

*The participating women received the following information about the preventive strategies:*

Four different strategies to prevent early-onset group B streptococcal disease in neonates.

1. The current treatment. All women with a fever, GBS bacteriuria or a previous child with GBS disease will be treated with intravenous antibiotics during labour. A maternal GBS swab will be taken in case of preterm birth or ruptured membranes for a period of more than 18 hours. If GBS colonisation is detected in the maternal swab, or if the gynaecologist thinks it is necessary, women will be treated with intravenous antibiotics.
2. The risk-based strategy. All women with one of the risk factors: preterm birth, ruptured membranes for a period of more than 18 hours, a previous child with GBS disease, GBS bacteriuria or a fever will be treated with intravenous antibiotics during labour.
3. The screening strategy. All women will be screened for GBS colonisation in the period between weeks 35 and 37 of pregnancy. Women with GBS colonisation will be treated with intravenous antibiotics during labour. Women for whom no test result is available will also be treated with intravenous antibiotics during labour.
4. The combination strategy. All women will be screened for GBS colonisation in the period between weeks 35 and 37 of pregnancy. Women with GBS colonisation AND a risk factor (preterm birth, ruptured membranes for a period of more than 18 hours, a previous child with GBS disease, GBS bacteriuria or fever) will be treated with intravenous antibiotics during labour. Women for whom no test result is available WITH a risk factor will also be treated with intravenous antibiotics during labour.

The interview questions were the following:

1. How do you feel about the different methods of detecting GBS colonisation and treatment using the screening strategy, the combination strategy, the risk-based strategy and the NVOG or Dutch guideline strategy? Which strategy do you prefer and why do you prefer it?
2. What do you see as the benefits and drawbacks of the different strategies? (*Consider: place of birth, swab taking, application of antibiotics while not being ill*)
3. Which information do you need to make an informed decision about the preventive strategies? (*Consider: your wishes regarding labour*)
4. How do you want to receive information about the prevention of EOGBS disease? (*Consider: leaflets, a consultation, by email, website and the person who informs you*)
